# Eyes that Do Not Meet the Eligibility Criteria of Clinical Trials on Age-Related Macular Degeneration: Proportion of the Real-World Patient Population and Reasons for Exclusion

**DOI:** 10.1155/2021/6635467

**Published:** 2021-04-17

**Authors:** Jae Hui Kim, Jong Woo Kim, Chul Gu Kim

**Affiliations:** Department of Ophthalmology, Kim's Eye Hospital, Seoul, Republic of Korea

## Abstract

**Background:**

To evaluate the proportion of eyes that do not meet the eligibility criteria of clinical trials on neovascular age-related macular degeneration (AMD) and the reasons for exclusion.

**Methods:**

This retrospective, observational study included 512 eyes of 463 patients diagnosed with treatment-naïve neovascular AMD. The proportion of eyes that did not meet the eligibility criteria of the Vascular Endothelial Growth Factor Trap-Eye: Investigation of Efficacy and Safety in Wet AMD (VIEW) studies were evaluated. The two most common reasons for exclusion were also evaluated in each subtype of neovascular AMD (typical neovascular AMD, polypoidal choroidal vasculopathy (PCV), and type 3 neovascularization).

**Results:**

Among the 512 eyes, 229 (44.7%) did not meet the eligibility criteria. In all the included eyes, the most common reasons for exclusion were good or poor visual acuity (169 eyes, 33.0%), followed by the presence of subretinal hemorrhage (47 eyes, 9.5%). Moreover, good or poor visual acuity was the most common reason for exclusion in all three subtypes of neovascular AMD. The second most common reason was a fovea-involving scar or fibrosis in typical neovascular AMD, subretinal hemorrhage in PCV, and other vascular diseases affecting the retina in type 3 neovascularization.

**Conclusions:**

Among the included cases, 44.7% did not meet the eligibility criteria for VIEW study, suggesting that the conclusion derived from clinical trials may not directly reflect the real-world outcomes. Additionally, the reasons for ineligibility differed among the different subtypes of neovascular AMD.

## 1. Introduction

Neovascular AMD is one of the primary causes of severe visual impairment in developed countries [1]. Previously, laser photocoagulation or photodynamic therapy was used as its mainstay treatment. However, the efficacy of these treatment modalities has obvious limitations. In 2006, the FDA approved anti-VEGF agent, ranibizumab, was introduced [2, 3], followed by aflibercept in 2012 [4]. In addition, the off-label use of bevacizumab [5] has been widely adopted. The introduction of these anti-VEGF agents has markedly improved the treatment outcomes of neovascular AMD, resulting in a significant decrease in the rate of visual loss and blindness [6]. Currently, clinical trials are actively being performed to develop better treatment methods for neovascular AMD.

In general, conducting clinical trials is expensive and requires years to complete [7, 8]. Therefore, estimating the time required to complete the planned patient enrollment is essential for establishing an efficient clinical trial plan. Clinical trials are usually conducted in a controlled environment and have sophisticated eligibility criteria. Thus, all patients cannot be enrolled into a trial, and some of them, or sometimes the majority of them, are excluded on the basis of the eligibility criteria.

Estimation of the proportion of patients in the study population who do not meet the eligibility criteria is important for several reasons. First, it may help to identify whether the results of the clinical trial can be applied to the real-world patients. Secondly, it may also help to predict the time required to finish the planned patient enrollment. In addition, if a particular set of criteria results in the exclusion of a relatively large number of patients, patient enrollment in future clinical trials could be accelerated by modifying some of these criteria. Furthermore, since the characteristics of neovascular AMD differ between Asian and Caucasian populations [9, 10], obtaining data on Asian populations would be of great value.

Therefore, the purpose of the present study was to evaluate the proportion of eyes that do not meet the eligibility criteria of clinical trials on neovascular AMD among the entire sample of eyes diagnosed with treatment-naïve neovascular AMD. The eligibility criteria of the VEGF Trap-Eye: Investigation of Efficacy and Safety in Wet AMD (VIEW) studies [4], were used for this investigation.

## 2. Materials and Methods

This retrospective, observational study was performed at a single center. The study was approved by the Institutional Review Board of Kim's Eye Hospital and was conducted in accordance with the tenets of the Declaration of Helsinki.

### 2.1. Patients

The study included consecutive patients diagnosed with treatment-naïve, active neovascular AMD between January 2017 and December 2017. Additionally, only patients aged ≥50 years who received intravitreal anti-VEGF injection after the diagnosis were included in this study.

### 2.2. Examinations and Image Analysis

Measurement of best-corrected visual acuity (BCVA) and a clinical examination using 90-diopter lens slit-lamp biomicroscopy were performed for all the patients, and intraocular pressure was measured using a noncontact tonometer. The fundus photographs were acquired using CX-1™ (Topcon, Tokyo, Japan). Optical coherence tomography (OCT) images were acquired using Spectralis HRA + OCT^®^ (Heidelberg Engineering GmbH, Heidelberg, Germany) or RS 3000^®^ (Nidek Co. Ltd. Tokyo, Japan). Fluorescein angiography and indocyanine green angiography (ICGA) images were also acquired using Spectralis HRA + OCT^®^ (Heidelberg Engineering GmbH). The size of the lesion was measured using fundus photographs and fluorescein angiography images.

The method of classification of neovascular AMD was similar to that used in our previous study [10]. The diagnosis of polypoidal choroidal vasculopathy (PCV) was based on the ICGA images [11, 12]. Type 3 neovascularization was diagnosed using a previously suggested method [13] and was identified on the basis of OCT and angiography results. Eyes not showing the characteristic features of PCV or type 3 neovascularization were classified as having typical neovascular AMD. Patients with definite chorioretinal anastomosis were classified as having typical neovascular AMD because it was uncertain whether the origin of choroidal neovascularization was the retina. If ICGA results were unavailable or if accurate classification was not possible, the patients were considered as unclassifiable. The ICGA images were analyzed by two independent examiners. In case of disagreement, the images were reexamined together and the disagreement was resolved by mutual discussion between the two examiners. Other images, including that of fluorescein angiography, OCT, and fundus photography, were evaluated by a single examiner (J.H.K.). The BCVAs were measured using decimal visual acuity chart and subsequently converted to logarithm of minimal angle of resolution (logMAR) value for further analysis.

### 2.3. Criteria Used for Analyses

The eligibility criteria of the VIEW studies [4] were classified into three categories as follows.

### 2.4. Category 1: Criteria that Could Not Be Accurately Assessed in the Present Study (Table 1)

The present study was a retrospective study based on the review of medical records. Thus, detailed evaluation of the patients' systemic or ocular conditions that are necessary in clinical trials were not routinely performed. Therefore, in case of some eligibility criteria, it was not possible to accurately assess whether the patients met the criteria or not. For example, most of the criteria regarding systemic conditions were included in category 1.

### 2.5. Category 2: Criteria Required for Inclusion in the Present Study (Table 2)

These included criteria that should be met in order for patients to be included in the present study, e.g., age and prior treatment criteria.

### 2.6. Category 3: Criteria Used for Result Analysis in the Present Study (Table 3)

These included criteria that were actually used for result analysis in the present study. If an eye did not meet all three inclusion criteria, or met at least one exclusion criterion, that eye was excluded from the clinical trial.

### 2.7. Analyses

The results of the examinations performed at diagnosis and the patients' medical history were carefully reviewed to identify the proportion of eyes that met each category 3 criterion. In addition, the proportion of eyes that met category 3 criteria among the three subtypes of neovascular AMD was compared. Thereafter, the proportion of eyes in the top two criteria met by the largest number of patients was compared between the different subtypes. Lastly, the top two criteria within each subtype group were presented.

For visual acuity analysis, the values of visual acuities indicated by the eligible criteria (Table 3) were converted into logMAR values as follows: 20/40 = 0.30 (logMAR) and 20/320 = 1.20 (logMAR). Thereafter, the logMAR values were used to assess whether visual acuities of the included patients met the eligible criteria.

### 2.8. Statistics

Statistical analyses were performed using SPSS for Windows/Macintosh, Version 12.0 (SPSS Inc., Chicago, IL, USA). The difference in proportion among the groups was analyzed using the chi-square test. *P* values less than 0.05 were considered significant.

## 3. Results

During the study period, 512 eyes of 463 patients (282 men and 181 women) were newly diagnosed with treatment-naïve neovascular AMD. The mean age of the patients was 70.0 ± 8.9 years. Among the eyes, 208 were classified as having typical neovascular AMD, 189 as having PCV, and 69 as having type 3 neovascularization. In the remaining 46 eyes, the subtype of neovascular AMD was unclassifiable. Demographic information of the patients is summarized in Table 4.

**Figure 1:**
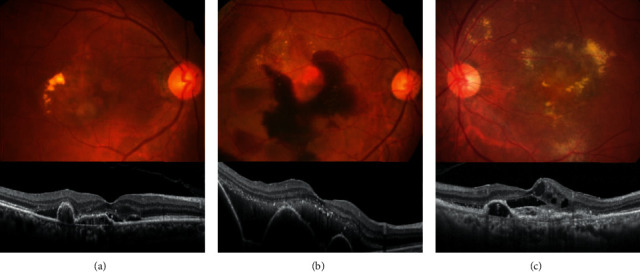
Fundus photography and optical coherence tomography findings of eyes which did not satisfy the eligible criteria. (a) Best-corrected visual acuity better than 20/40, (b) fovea-involving large subretinal hemorrhage, (c) fovea-involving scar.

Among the 512 eyes, 229 (44.7%) did not satisfy at least one inclusion or exclusion category 3 criterion (Table 3): 171 eyes (33.4%) did not satisfy one criterion, 39 (7.6%) did not satisfy two criteria, and 19 (3.7%) did not satisfy three or more criteria.  Figure 1 shows representative cases of eyes did not satisfy the eligible criteria. Among the category 3 criteria, the top two criteria not met by the patients were the visual acuity criterion (Table 3, #3) and submacular hemorrhage criterion (Table 3, #5). One hundred sixty-nine eyes (33.0%) did not meet the visual acuity criterion; the logMAR BCVA at diagnosis was better than 0.30 (Snellen equivalents, 20/40) in 95 eyes and worse than 1.20 (Snellen equivalents, 20/320) in 74 eyes. Forty-seven eyes (9.2%) did not meet the subretinal hemorrhage criterion.

A comparison among the subtypes of neovascular AMD (*N* = 466) showed that 102 eyes (49.0%) in the typical neovascular AMD group, 89 in the PCV group (47.1%), and 23 (33.3%) in the type 3 neovascularization group did not meet at least one category 3 criterion (Table 5). No difference was observed in the proportion of eyes among the three subtypes (*P*=0.070). In the analysis including the top two most commonly not met criteria, the visual acuity criterion (category 3 criterion #3) was not satisfied in 80 eyes (38.5%) in the typical neovascular AMD group, 60 (31.7%) in the PCV group, and 17 (24.6%) in the type 3 neovascularization group. The incidence was not significant among the three subtypes (*P*=0.083). On more specific analysis, there was a significant difference in the number of eyes that showed logMAR BCVA better than 0.30 (*P*=0.032): 40 eyes (19.2%) in the typical neovascular AMD group, 44 eyes (23.3%) in the PCV group, and 6 eyes (8.7%) in the type 3 neovascularization group. In addition, there was a significant difference in the incidence of eyes that showed logMAR BCVA worse than 1.20 (*P*=0.009): 40 eyes (19.2%) in the typical neovascular AMD group, 16 eyes (8.5%) in the PCV group, and 11 eyes (15.9%) in the type 3 neovascularization group. The submacular hemorrhage criterion (category 3 criterion #5) was not satisfied in 9 eyes (4.3%) in the typical neovascular AMD group, 31 (16.4%) in the PCV group, and 2 (2.9%) in the type 3 neovascularization group. A significant difference was observed in the proportion of eyes among the three subtypes (*P* < 0.001).

In the typical neovascular AMD group, the most common unmet criterion was the visual acuity criterion (criterion #3; 80 eyes, 38.5%), followed by the scar, fibrosis, or atrophy involving the center of the fovea criterion (criterion #7; 11 eyes, 5.3%). In the PCV group, the most common criterion not met was the visual acuity criterion (criterion #3; 60 eyes, 31.7%), followed by the subretinal hemorrhage criterion (criterion #5; 31 eyes, 16.4%). In the type 3 neovascularization group, the most common criterion not met was the visual acuity criterion (criterion #3; 17 eyes, 24.6%), followed by the other vascular diseases affecting the retina criterion (criterion #9; 3 eyes, 4.3%).

## 4. Discussion

Although the VIEW study was performed nearly 10 years ago, the VIEW study criteria were used in the present study because aflibercept is one of the most widely used drugs to treat neovascular AMD. In the present study, 44.7% of the patients newly diagnosed with treatment-naïve neovascular AMD in the real-world setting did not meet the eligibility criteria which were similar to that of the VIEW studies. That is, if a clinical trial using these eligibility criteria is performed at our institution, 44.7% of patients will not pass the screening. However, the remaining 55.3% could be potential candidates for the clinical trial.

More importantly, the efficacy of the drug could not be tested in 44.7% of the patients in the clinical trial suggesting that the treatment efficacy of the drug is unknown in those patients. For this reason, further studies are required to evaluate the efficacy of the drug in patients with certain characteristics that were excluded from clinical trials. For example, since patients with submacular hemorrhages were excluded from the VIEW studies [4], further clinical trial was performed to evaluate the efficacy of aflibercept in this condition [14]. In fact, the treatment outcomes were somewhat different between the clinical trials [4] and real-world data [15]. Different patients' characteristics between the two different conditions may have an influence on this difference.

The most common criterion that resulted in the exclusion of the largest number of eyes was the visual acuity criterion; only eyes with an Early Treatment Diabetic Retinopathy Study (ETDRS) BCVA of 20/40 to 20/320 (letter score of 73 to 25) could be included. A similar visual acuity criterion was also used in other key clinical trials on neovascular AMD [2, 16, 17]. In the present study, 169 eyes did not satisfy this criterion, accounting for 33.0% of the entire study population and approximately 73.8% of the excluded cases. The second most common criterion was the subretinal hemorrhage criterion. If an eye exhibited subretinal hemorrhage that affected either 50% or more of the total lesion area, or if blood was observed under the fovea and was 1 or more disc areas in size, that eye was excluded from the study. In the present study, 47 eyes exhibited submacular hemorrhage that satisfied this criterion. This accounted for 9.2% of the entire study population and approximately 20.5% of the excluded cases.

An interesting finding of the present study was that the reasons for exclusion differed according to the subtypes of neovascular AMD. In all three subtypes, the largest number of eyes was excluded by the visual acuity criterion. The proportion of excluded eyes was relatively higher in the typical neovascular AMD group (38.5%) than in the PCV group (31.7%) or the type 3 neovascularization group (24.6%). In addition, the primary reason for exclusion differed among the three groups. Particularly, in the PCV group, majority of eyes were excluded because of good visual acuity, and only a small proportion of eyes were excluded because of poor visual acuity.

A significant difference was observed in the proportion of eyes excluded because of the subretinal hemorrhage criterion. The proportion was markedly higher in the PCV group (16.4%) than in the typical neovascular AMD group (4.3%) or the type 3 neovascularization group (2.9%). When all three groups were analyzed together, submacular hemorrhage was the second most common reason for exclusion. However, when each subtype was analyzed separately, the results changed. In the typical neovascular AMD and type 3 neovascularization groups, only a small proportion of eyes was excluded by the subretinal hemorrhage criterion.

The present study may provide some useful information when planning a clinical trial for drug development. First, the study provides the number and proportion of patients who can be candidates for a clinical trial from among the entire real-world patient population. Although several factors, such as informed consent or detailed systemic conditions, could not be evaluated, our data can be used to obtain a rough estimate of how many patients can be enrolled in a clinical trial. Second, our study shows which parts of the eligibility criteria should be modified to facilitate patient enrollment. For example, patient enrollment can be facilitated by broadening the range of visual acuity. In a PCV study, including some hemorrhage cases by modifying the eligibility criterion regarding hemorrhage may be helpful in accelerating patient enrollment.

Each clinical trial has its own inclusion and exclusion criteria. Thus, treatment outcomes in a certain condition that does not meet the eligibility criteria cannot be fully evaluated through the trial. In such cases, subsequent studies are required to verify it. The results of the present study may also provide useful information when planning these subsequent studies. For instance, 18.6% of patients can be potential candidates when planning a study on patients with initial visual acuity better than 20/40. In addition, the proportion will be 9.2% when planning a study on patients with subretinal hemorrhage.

In general, clinical trials are expensive [7, 8], and this is an important burden to the company or organization hosting the trial [18]. In addition, the increase in the cost of drug development may increase the price of the drug and eventually increase the socioeconomic burden. To prevent unexpected increase in the cost of undertaking clinical trials, it is important to select suitable institutions for the trials. If information such as the results presented in this study is available to many institutions worldwide and is easily accessible to the public, it will be a great help to global pharmaceutical companies or research institutes to easily identify the characteristics of institutions located in various countries. As a result, such information will contribute to the selection of more suitable institutions for clinical trials. In future, the development of artificial intelligence-assisted prescreening of participants eligible for clinical trial [19] may help facilitate this process.

To our knowledge, only two previous studies have investigated the proportion of patients that do not meet the eligibility criteria of clinical trials on neovascular AMD [20, 21]. George et al. found that less than half of the real-world patients would have passed the angiographic eligibility criteria used in the MARINA (Minimally Classic/Occult Trial of the Anti-VEGF Antibody Ranibizumab in the Treatment of Neovascular AMD) and ANCHOR (Anti-VEGF Antibody for the Treatment of Predominantly Classic Choroidal Neovascularization in AMD) studies. [20] The main reasons were “choroidal neovascularization [CNV] < 50% of the lesion” in 33%, “location of CNV (extrafoveal, juxtafoveal, or peripapillary)” in 23.1%, and “size > 12 disc areas and blood > 50%” in 2.3% [20]. In the study of Gilles et al. [21], among 646 eyes that were treated in real-world setting, 401 (62.1%) met the eligible criteria for MARINA study. A substantial number of eyes failed to satisfy the criteria due to good or poor visual acuity [21].

Although the method of analysis was similar between the present study and the study by George et al., there were obvious differences between the two studies. First, the primary purpose of the study by George et al. was to determine the proportion of patients who may be eligible for treatment with ranibizumab [20], whereas the primary purpose of the present study was more focused on the clinical trials themselves. Therefore, more detailed evaluation of each eligibility criterion was performed in the present study. Second, the reason for ineligibility differed between the two studies. In the study by George et al., only a small proportion of patients was found to be ineligible because of the observation of blood [20], whereas it was a primary reason for exclusion in the present study. We believe this difference could be attributed to the ethnic differences between the patient populations included in the two studies; accordingly, the proportion of PCV might be markedly higher in the present study on an Asian population.

It is important to know whether the drug efficacy shown in clinical trials is also valid in real-world setting, especially in patients excluded from the trials. In general, the real-world outcomes are relatively comparable or slightly inferior to that reported in clinical trials [15, 22, 23]. Woo et al. reported 12-week visual outcomes of ranibizumab based on real-world, postmarketing surveillance study data [22]. In that study, the visual gain was 7.5 letters at 12 weeks in treatment-naïve patients and this was comparable to that reported in previous clinical trials [2, 4, 5]. More recently, Eter et al. reported a 24-month real-world efficacy of aflibercept [15]. In that study, 6.7 letters of visual improvement were noted at 24 months in patients who received regular treatment. Altaweel et al. evaluated treatment outcomes of eyes with lesions composed of >50% blood, based on comparison of age-related macular degeneration treatment trials (CATT) data [23]. Patients with this characteristic were excluded from early clinical trials for ranibizumab [2]. As a result, eyes with the blood showed similar outcomes when compared to eyes without the blood. Based on this finding, the authors concluded that eyes with neovascular AMD lesions composed of >50% blood can be managed clinically in a similar manner as those without it [23]. However, in some cases, somewhat different outcomes are noted in real-world patients. In the study of Jung et al. [24], vitrectomized eyes required more frequent injections than nonvitrectomized eyes, suggesting patients' characteristics can influence the treatment outcomes. These results suggest that the drugs introduced based on the results of clinical trials can be prescribed to real-world patients, but with care.

This study has some limitations. This was a retrospective study performed in a single center. Thus, the included patients may not be representative of the entire global population. Moreover, not all the eligibility criteria were evaluated. In addition, all the included patients were Korean. The subtypes of neovascular AMD differ among different ethnic groups. In particular, PCV is more prevalent in Asian populations than in Caucasian populations [9]. Thus, the results of the present study may not be valid in other ethnic groups. Since the eligibility criteria of the VIEW studies were used, the result may change when using the eligibility criteria of different studies. Visual acuities were measured using the decimal visual acuity chart and equivalent logMAR values were used for analysis. Thus, the result may differ when using the Snellen or ETDRS chart. The size of the lesion was measured using fundus photographs and fluorescein angiography images. Thus, shallow retinal pigment epithelial detachments or shallow subretinal fluid may not be included when determining the lesion size. If these lesions are included in the lesion size, the proportion of patients who did not meet the lesion size criteria may change. Lastly, in the present study, ICGA was performed in most of the patients, and the result in PCV was analyzed separately. Although this approach can be appropriate for Asian patients, there is a doubt as to whether this approach is appropriate for other ethnic groups. In addition, part of our results cannot be utilized when ICGA examination is not performed.

In summary, application of eligibility criteria similar to that of the VIEW studies resulted in 44.7% of the study patients ineligible for at least one inclusion or exclusion criterion. The most common reason for exclusion was that the visual acuity of the patients was beyond the range of the inclusion criterion. In addition, the reason for exclusion differed among the different subtypes of neovascular AMD. Given the current trend of increasing numbers of global clinical trials, selecting the appropriate institutions for conducting clinical trials based on accurate information will be very important. We believe that our findings would be useful when designing future clinical trials on Asian patients with neovascular AMD. Additional multicenter studies with larger study population and more standardized visual acuity measurement are needed to accurately evaluate the proportion and characteristics of the real-world patients who do not meet the criteria for clinical trials.

## Figures and Tables

**Table 1:** Eligibility criteria of the VEGF Trap-Eye: Investigation of Efficacy and Safety in Wet AMD studies that could not be accurately assessed in the present study (category 1).

Criterion
*Inclusion Criteria*
1) Willing, committed, and able to return for ALL clinic visits and complete all study related procedures.
2) Able to read (or, if unable to read due to visual impairment, be read to verbatim by the person administering the informed consent or a family member) and understand and willing to sign the informed consent form.
3) Signed informed consent form.

*Exclusion Criteria*
4) Significant media opacities, including cataract, in the study eye that might interfere with visual acuity, assessment of safety, or fundus photography.
5) Any concurrent ocular condition in the study eye which, in the opinion of the investigator, could either increase the risk to the patient beyond what is to be expected from standard procedures of intraocular injection or which otherwise may interfere with the injection procedure or with evaluation of efficacy or safety.
6) History of any vitreous hemorrhage within 4 weeks prior to Visit 1 in the study eye.
7) Any ocular or periocular infection within the last 2 weeks prior to screening in either eye.
8) Any history of uveitis in either eye.
9) Presence or history of scleromalacia in either eye.
10) Previous therapeutic radiation in the region of the study eye.
11) History of other diseases, metabolic dysfunction, physical examination finding, or clinical laboratory finding giving reasonable suspicion of a disease or condition that contraindicates the use of an investigational drug or that might affect interpretation of the results of the study or render the patient at high risk for treatment complications.
12) Participation as a patient in any clinical study within the 12 weeks prior to day 1.
13) Any systemic or ocular treatment with an investigational agent in the past 12 weeks prior to day 1.
14) The use of long acting steroids, either systemically or intraocularly, in the 6 months prior to day 1.
15) Any history of allergy to povidone iodine.
16) Presence of any contraindications indicated in the FDA approved label for ranibizumab (Lucentis®; Genentech Inc., South San Francisco, CA).
17) Females who are pregnant, breastfeeding, or of childbearing potential, unwilling to practice adequate contraception throughout the study. Adequate contraceptive measures include oral contraceptives (stable use for 2 or more cycles prior to screening); IUD; Depo-Provera® (Pfizer, Inc. New York); Norplant® System (Pfizer, Inc. New York) implants; bilateral tubal ligation; vasectomy; condom or diaphragm plus either contraceptive sponge, foam, or jelly.

VEGF = vascular endothelial growth factor, AMD = age-related macular degeneration.

**Table 2:** Eligibility criteria of the VEGF Trap-Eye: Investigation of Efficacy and Safety in Wet AMD studies required for inclusion in the present study (category 2).

Criterion
*Inclusion Criteria*
1) Men and women ≥50 years of age.

*Exclusion Criteria*
2) Any prior ocular (in the study eye) or systemic treatment or surgery for neovascular AMD except dietary supplements or vitamins.
3) Any prior or concomitant therapy with another investigational agent to treat neovascular AMD in the study eye, except dietary supplements or vitamins.
4) Prior treatment with anti-VEGF agents.
5) Known serious allergy to the fluorescein sodium for injection in angiography.

Abbreviations: VEGF = vascular endothelial growth factor, AMD = age-related macular degeneration.

**Table 3:** Eligibility criteria of the VEGF Trap-Eye: Investigation of Efficacy and Safety in Wet AMD studies used for result analysis (category 3) and the number of eyes that did not meet the inclusion criteria or that met the exclusion criteria.

Characteristic	Total (*N* = 512)
*Inclusion Criteria*	
1) Active primary subfoveal CNV lesions secondary to AMD, including juxtafoveal lesions that affect the fovea as evidenced by FA in the study eye.	8 (1.6%)^*∗*^
2) CNV must be at least 50% of total lesion size.	25 (4.9%)^*∗*^
3) ETDRS best-corrected visual acuity of 20/40 to 20/320 (letter score of 73 to 25) in the study eye.	169 (33.0%)^*∗*^

*Exclusion Criteria*	
4) Total lesion size > 12 disc areas (30.5 mm^2^), including blood, scars, and neovascularization as assessed by FA in the study eye.	13 (2.5%)^†^
5) Subretinal hemorrhage that is either 50% or more of the total lesion area, or if the blood is under the fovea and is 1 or more disc areas in size in the study eye. (If the blood is under the fovea, then the fovea must be surrounded 270° by visible CNV.)	47 (9.2%)^†^
6) Scar or fibrosis, making up > 50% of total lesion in the study eye.	9 (1.8%)^†^
7) Scar, fibrosis, or atrophy involving the center of the fovea in the study eye.	12 (2.3%)^†^
8) Presence of retinal pigment epithelial tears or rips involving the macula in the study eye.	2 (0.4%)^†^
9) History or clinical evidence of diabetic retinopathy, diabetic macular edema, or any other vascular disease affecting the retina, other than AMD, in either eye.	15 (2.9%)^†^
10) Any concurrent intraocular condition in the study eye (e.g., cataract) that, in the opinion of the investigator, could require either medical or surgical intervention during the 96-week study period.	4 (0.8%)^†^
11) Prior vitrectomy in the study eye.	2 (0.4%)^†^
12) Any history of macular hole of stage 2 and above in the study eye.	0^†^
13) Any intraocular or periocular surgery within 3 months of day 1 on the study eye, except lid surgery, which may not have taken place within 1 month of day 1, as long as it is unlikely to interfere with the injection.	0^†^
14) Prior trabeculectomy or another filtration surgery in the study eye.	0^†^
15) Uncontrolled glaucoma (defined as intraocular pressure ≥ 25 mmHg despite treatment with antiglaucoma medication) in the study eye.	2 (0.4%)^†^
16) Active intraocular inflammation in either eye.	0^†^
17) Active ocular or periocular infection in either eye.	0^†^
18) Aphakia or pseudophakia with absence of posterior capsule (unless it occurred as a result of an yttrium aluminum garnet (YAG) posterior capsulotomy) in the study eye.	0^†^
19) History of corneal transplant or corneal dystrophy in the study eye.	0^†^

VEGF = vascular endothelial growth factor, AMD = age-related macular degeneration, CNV = choroidal neovascularization, FA = fluorescein angiography, ETDRS = Early Treatment Diabetic Retinopathy Study. ^*∗*^: Number of eyes that did not meet the inclusion criteria. †: Number of eyes that met the exclusion criteria.

**Table 4:** Demographic information of 463 patients (512 eyes).

Characteristics	
Age, years	70.0 ± 8.9
Sex, men: women	282 (60.9%): 181 (39.1%)
Diabetes mellitus	101 (21.8%)
Hypertension	225 (43.9%)
Subtype of neovascular AMD	
Typical neovascular AMD	208 (40.7%)
Polypoidal choroidal vasculopathy	189 (36.9%)
Type 3 neovascularization	69 (13.5%)
Unclassifiable	46 (8.9%)
Baseline best-corrected visual acuity, logMAR	0.69 ± 0.54

Data are presented as mean ± standard deviation or No. (%), when applicable. Abbreviations: AMD, age-related macular degeneration; logMAR, logarithm of minimal angle of resolution.

**Table 5:** Comparison of the proportion of eyes that did not meet category 3 criteria among the three subtypes of neovascular age-related macular degeneration.

Characteristic	Typical neovascular AMD (*N* = 208)	PCV (*N* = 189)	Type 3 neovascularization (*N* = 69)	*P* value^*∗*^
Eyes that did not meet at least one category 3 criterion	103 (49.5%)	89 (47.1%)	22 (31.9%)	0.036
Visual acuity criterion	80 (38.5%)	60 (31.7%)	17 (24.6%)	0.083
Visual acuity better than 20/40	40 (19.2%)	44 (23.3%)	6 (8.7%)	0.032
Visual acuity worse than 20/320	40 (19.2%)	16 (8.5%)	11 (15.9%)	0.009
Submacular hemorrhage criterion	9 (4.3%)	31 (16.4%)	2 (2.9%)	<0.001

Data are presented as number (%).AMD = age-related macular degeneration, PCV = polypoidal choroidal vasculopathy. ^*∗*^Statistical analysis was performed using the chi-square test.

## Data Availability

Data are available on request.
